# Discovery in Genetic Skin Disease: The Impact of High Throughput Genetic Technologies

**DOI:** 10.3390/genes5030615

**Published:** 2014-08-04

**Authors:** Thiviyani Maruthappu, Claire A. Scott, David P. Kelsell

**Affiliations:** Centre for Cutaneous Research, Blizard Institute, Barts and The London School of Medicine and Dentistry, Queen Mary University of London, 4 Newark Street, London E1 2AT, UK; E-Mails: t.maruthappu@qmul.ac.uk (T.M.); c.a.scott@qmul.ac.uk (C.A.S.)

**Keywords:** genodermatoses, exome, ichthyosis, atopic eczema, psoriasis

## Abstract

The last decade has seen considerable advances in our understanding of the genetic basis of skin disease, as a consequence of high throughput sequencing technologies including next generation sequencing and whole exome sequencing. We have now determined the genes underlying several monogenic diseases, such as harlequin ichthyosis, Olmsted syndrome, and exfoliative ichthyosis, which have provided unique insights into the structure and function of the skin. In addition, through genome wide association studies we now have an understanding of how low penetrance variants contribute to inflammatory skin diseases such as psoriasis vulgaris and atopic dermatitis, and how they contribute to underlying pathophysiological disease processes. In this review we discuss strategies used to unravel the genes underlying both monogenic and complex trait skin diseases in the last 10 years and the implications on mechanistic studies, diagnostics, and therapeutics.

## 1. Introduction

The advent of high throughput single nucleotide polymorphism (SNP) genotyping and latterly, next generation sequencing (NGS) technology including whole exome sequencing (WES) have revolutionised our approach to genetic diagnostics and novel gene discovery in the genodermatoses—a group of inherited skin disorders.

Prior to this, technologies including linkage analysis using genome wide microsatellite panels in combination with candidate gene screening by PCR and Sanger sequencing have been the primary method for discerning new skin disease-associated loci. Successes with this approach include Hailey-Hailey Disease (OMIM #169600) [1], Netherton Syndrome (OMIM #256500) [2], Darier-Disease (OMIM #124200) [3], and Dyschromatosis symmetrica hereditaria (OMIM #127400) [4]. Candidate gene screening approaches have also yielded success, particularly in deciphering the keratin disorders [5]. However, clinical and likely genetic heterogeneity of skin diseases and the availability of DNA from probands only, or from small families, have hindered disease gene discovery for many disorders [6]. This can now be surmounted with high-density SNP homozygosity mapping for consanguineous recessive disorders, and in particular NGS and WES for dominant and recessive disorders, which has facilitated our understanding of some of the genetic make up of common diseases.

Skin diseases are ideal for determining genotype-phenotype correlations because of the relative ease with which clinical and histological examination can be made. In addition, inflammatory pathways involved in the pathogenesis of skin diseases such as psoriasis vulgaris (PV) are relevant to a number of other immune-mediated diseases including inflammatory bowel disease and rheumatoid arthritis [7].

The genetic bases of many monogenic skin diseases have been unravelled and in this review we focus on examples of discoveries in cutaneous genetics, applying different strategies such as SNP microarray, microsatellite linkage analysis, targeted NGS and WES. Equally, it has also been informative in understanding the significance of *de novo* mutations including the unusual phenomenon of revertant mosaicism in the skin, where spontaneous correction of a disease-causing mutation in a somatic cell occurs [8]. We have also gained insights into complex trait diseases and will explore what contributions these have made to mechanistic insights, diagnosis and treatment of common skin diseases including psoriasis, atopic dermatitis (AD) (eczema), and skin cancer.

## 2. Harlequin Ichthyosis

The discovery that *ABCA12* gene mutations are associated with the skin disease harlequin ichthyosis (HI) is an example of where SNP microarray technology was used successfully to elucidate the genetic locus associated with this disease [9].

The inherited ichthyoses are a heterogeneous group of disorders characterised by skin scaling, often of the whole surface, and hyperkeratosis [10]. Syndromic (affecting multiple tissues) as well as nonsyndromic forms of ichthyosis exist and mutations in multiple genes are associated with disease including *TGM1* (OMIM *190195), *NIPAL4* (OMIM *609383), *STS* (OMIM *300747), *ALOX12B* (OMIM *603741), *ALOXE3* (OMIM *607206), *CYP4F22* (OMIM *611495), and *FLG* (OMIM *135940) amongst others (reviewed in [10]). Autosomal recessive congenital ichthyosis (ARCI) is comprised of three main groups: congenital ichthyosiform erythroderma (CIE), lamellar ichthyosis (LI) and HI [10]. HI (OMIM #242500) is the most severe form of ichthyosis and has a high perinatal mortality, with babies presenting at birth with hard scale plates with deep fissures, eclabium, and bilateral ectropion (reviewed in [9,11]).

The discovery of the genetic cause of HI was hampered by availability of DNA from only affected family members or from small families due to the severity of the condition, thus genetic linkage studies were unfeasible [9]. To investigate the genetic basis of HI, Kelsell *et al.* (2005) [9] used a SNP microarray to map a block of homozygosity on chromosome 2q35 and to identify a minimal region between HI patients from consanguineous parents, which contained the *ABCA12* gene. ABCA12 belongs to the ATP-binding cassette (ABC) A family of transporters, some members of which have been implicated in lipid transport (reviewed in [12]).

*ABCA12* was a promising gene candidate for HI because patient skin displayed aberrant lipid distribution [9] and missense mutations in *ABCA12* were already known to be associated with another form of ARCI, LI [13]. PCR and Sanger sequencing of the *ABCA12* gene in HI patients confirmed that recessive mutations were associated with HI [9,14]. Mutations in *ABCA12* are now known to be associated with all three forms of ARCI (reviewed in [10]). However, unlike for LI and CIE, in which largely missense *ABCA12* mutations are associated with disease [13,15,16], HI is usually associated with loss of function gene mutations including nonsense, frameshift, and splice site mutations, which severely disrupt the cellular functions of ABCA12 [9,17,18,19]. However, there are reports of patients who have ABCA12 missense mutations [9,11,18,20,21,22]. HI patients with homozygous loss of function mutations have an increased risk of mortality, indicating a survival advantage for patients with compound heterozygous mutations [11].

ABCA12 is thought to transport lipids via lamellar granules where they are processed and released to form lipid lamellae constituting the stratum corneum in the epidermis [14,23]. A reduction in the number, and structural abnormalities, of lamellar granules has been observed in HI patient skin [14,24,25]. In addition, characterisation of HI patient skin has shown a loss of nonpolar lipids [26] and abnormal glucosylceramide localization [14], and experiments with patient-derived keratinocytes showed aberrant glucosylceramide accumulation in lamellar granules [27], which is indicative of a lipid transport defect as a result of loss of ABCA12 function [14,26,27].

Similarly, *Abca12* knockout mice models [28,29,30] and an *abca12* knockout zebrafish model [31] showed features of aberrant lipid transport compared to controls (reviewed in [32]). HI skin also shows features of premature terminal differentiation and a decreased expression of certain proteases, which suggests that loss of ABCA12 disrupts keratinocyte differentiation and epidermal desquamation, resulting in the formation of an aberrant epidermal barrier [26].

Prior to the discovery of the genetic cause of HI, prenatal diagnostic investigations depended on obtaining a foetal biopsy for analysis by electron microscopy, and on sonography [33,34] (reviewed in [35]). The discovery of the genetic cause of different ichthyoses, including HI, represents a major milestone in the ability to perform genetic diagnosis, carrier screening, genetic counselling, and prenatal diagnosis.

Current approaches to genetic screening for HI can involve screening specific exons, as there are some recurrent ethnic group mutations in *ABCA12* [18,19] and using WES, circumventing the need for performing PCR and Sanger sequencing of all 53 coding exons of the *ABCA12* gene.

## 3. Exfoliative Ichthyosis

The discovery of cystatin A (*CSTA*) gene mutations in association with exfoliative ichthyosis [36] is an example of the successful implementation of combining SNP microarray analysis with targeted NGS to determine the genetic cause of disease.

Autosomal recessive exfoliative ichthyosis (OMIM #607936) is characterised by palmoplantar skin peeling and dry scaly skin, with trauma and moisture aggravating the condition [36]. Microsatellite linkage analysis of two related Bedouin families initially suggested linkage of the disease to chromosome 12q13, which contains the type II keratin cluster [37].

Blaydon *et al.* (2011) [36] revisited this family and applied whole genome homozygosity mapping which revealed a common block of homozygosity between affected Bedouin patients on chromosome 3q21 as the likely disease gene location. Sequence capture and NGS of this region was then performed and revealed a splice site mutation in *CSTA*, which was found to segregate with exfoliative ichthyosis in the Bedouin family. This locus was missed in the microsatellite genome scans performed by Hatsell *et al.* (2003) [37] due to markers for this region being uninformative. Sanger sequencing of *CSTA* in a different family with exfoliative ichthyosis revealed a homozygous nonsense mutation which also segregated with disease [36]. In a subsequent study, WES revealed a novel homozygous nonsense mutation in *CSTA* in a large family with acral peeling skin syndrome [38] with similar clinical features to the patients reported in Blaydon *et al.* (2011) [36].

Cystatins are cysteine protease inhibitors which are thought to have a protective function against endogenous and external proteases, and to potentially modulate the degradation of intra- and extracellular proteins (reviewed in [39]). CSTA has been identified as a constituent of the cornified envelope [40] and is expressed in the suprabasal layers of the epidermis, the highest expression of which is in the granular layer [36,41]. CSTA is secreted by keratinocytes *in vitro* and has also been found in sweat, and is believed to have a protective role by inhibiting the proteolytic activity of dust mite allergens Der p 1 and Der f 1 [42]. CSTA levels have also been implicated as prognostic markers in different cancers [43,44,45].

Characterisation of skin from exfoliative ichthyosis patients with *CSTA* mutations revealed widened intercellular gaps in the lower epidermis, whereas the upper epidermal layers appeared normal with no evident barrier defect [36]. Experiments using an *in vitro* keratinocyte cell knockdown model showed an adhesion defect in response to mechanical stress, and an organotypic *CSTA* knockdown model showed similar abnormalities to the patient skin [36]. This finding is indicative of CSTA having a key role in keratinocyte adhesion in the basal epidermal layers and that loss of CSTA causes a predisposition to epidermal splitting. There were no obvious abnormalities in a murine model with a chromosomal deletion, which included the *Csta* gene [46], although investigation of a skin phenotype was not described.

## 4. Olmsted Syndrome

The genetic basis of various skin diseases (Table 1) has been determined using exome sequencing technology. One example where WES enabled the identification of the underlying causative genes is Olmsted syndrome (OS) [47,48]. OS (OMIM #614594) is a rare disorder characterised by mutilating palmoplantar keratoderma and periorificial keratosis. Additional clinical features include constriction of the digits, dystrophy of the nails, diffuse alopecia and a predisposition to infection and development of squamous cell carcinoma on keratotic lesions [47]. Different modes of inheritance have been hypothesised [47,48,49,50].

**Table 1:** Examples of genes associated with skin disease discovered using exome sequencing technology.

Gene	Disease	Mode of Inheritance	Reference
*AAGAB*	Punctate palmoplantar keratoderma Type I	AD	[51,52]
*ADAM10*	Reticulate acropigmentation of Kitamura	AD	[53]
*AQP5*	Nonepidermolytic palmoplantar keratoderma	AD	[54]
*ENPP1*	Cole disease	AD	[55]
*EXPH5*	Inherited skin fragility	AR	[56]
*HOXC13*	Pure hair and nail ectodermal dysplasia	AR	[57]
*KANK2*	Palmoplantar keratoderma and woolly hair	AR	[58]
*MBTPS2*	Olmsted syndrome	XLR	[48]
*POFUT1*	Dowling-Degos disease	AD	[59]
*POGLUT1*	Dowling-Degos disease	AD	[60]
*SERPINB7*	Nagashima-type palmoplantar keratosis	AR	[61]
*TRPV3*	Olmsted syndrome	AD/AR	[47]/[62]

AD: autosomal dominant; AR: autosomal recessive; XLR: X-linked recessive.

WES was used successfully to identify mutations in the Transient Receptor Potential Cation Channel, Subfamily V, Member 3 (*TRPV3*) gene [47], and the Membrane-Bound Transcription Factor Protease, Site 2 (*MBTPS2*) gene [48] to be associated with OS.

Lin *et al.* (2012) performed WES of an OS patient and her unaffected parents and identified a novel *de novo* heterozygous mutation p.G573S in *TRPV3* [47]. Screening for *TRPV3* mutations in five other OS patients revealed that three were heterozygous for p.G573S, one heterozygous for p.G573C and one heterozygous for p.W692G [47].

TRPV3 is a member of the TRPV cation channel family, and is known to be expressed in various tissue types including skin and hair follicles [63,64,65]. The murine *TRPV3* mutants p.G573S and p.G573C were discovered in spontaneous hairless rodent strains that develop dermatitis, a trait inherited in an autosomal dominant manner [66]. *Trpv3* knockout mice display wavy hair, curly whiskers and a defective skin barrier, and it is believed that TRPV3 associates with TGF-α/EGFR in a signalling pathway to modulate keratinocyte differentiation and hair morphogenesis [67].

*In vitro* functional studies with the three OS-associated TRPV3 mutants indicated that they are gain of function mutants, creating constitutively open channels and causing increased cell death of cells expressing the mutants [47]. Similar results were obtained in *in vitro* expression studies with the murine TRPV3 mutants p.G573S and p.G573C [68]. It has been hypothesised that *in vivo* the mutants may cause apoptosis and subsequent keratoderma in patients, and could contribute to their pruritis [47].

A subsequent study using WES revealed the recurrent *TRPV3* mutation p.G573S in sporadic OS [69]. Screening by Sanger sequencing has also revealed a homozygous mutation in an OS patient, indicating recessive inheritance [62]. Both recessive [70] and sporadic [71] *TRPV3* mutations have been associated with atypical OS with erythromelalgia.

Exome sequencing of two affected males reported previously in a consanguineous pedigree [72] in which OS followed a suggested X-linked recessive inheritance pattern, revealed a novel *MBTPS2* gene mutation which segregated with disease in the family [48]. This discovery expands the number of disorders attributed to *MBTPS2* gene mutations, as other mutations in this gene are associated with ichthyosis follicularis with atrichia and photophobia (IFAP) syndrome [73,74,75], BRESEK/BRESHECK syndrome [76], and keratosis follicularis spinulosa decalvans (KFSD) [77].

MBTPS1 and MBTPS2 are involved in activating signalling proteins such as the transcription factors SREBPS, enabling cells to respond to sterols [78,79] and in the processing of ATF6, which is a component of the unfolded protein response (UPR) [80]. *In vitro* functional studies with IFAP and KFSD MBTPS2 mutants revealed decreased sterol responsiveness compared to wild-type [73,77], and mutants which caused the greatest impairment of enzyme activity seemed to be associated with increased disease severity in patients [73].

## 5. Complex Traits of the Skin

In the last 10 years there have been landmark discoveries in our understanding of the genetic basis and pathophysiology of inflammatory skin diseases, most notably PV and AD. Both are common, complex diseases, in which a host of environmental factors can trigger disease in genetically susceptible individuals [81,82]. Inflammatory dermatoses are associated with both a significant burden on healthcare resources and patients’ quality of life [83,84].

Identification of susceptibility loci for PV and AD have resulted from developments in genome wide association studies (GWAS), which have been applied to all common disorders. Information has been generated by the HapMap and 1000 Genomes projects, in parallel with the technology to genotype multiple individual DNA samples at one million or more loci, allowing SNPs to be reviewed and enabling comparisons of allele frequency between large numbers of cases and controls to identify those which confer risk of disease [85]. The development of DNA microarray based genotyping allows up to a million SNPs to be tested simultaneously.

## 6. Psoriasis

PV is a common and chronic inflammatory disease, which can affect the skin, nails and joints. It is characterised by immune-mediated epidermal hyperproliferation [86]. It is a highly heritable disease, with increased concordance in monozygotic *versus* dizygotic twins (65%–72% *versus* 15%–30% respectively) [87]. During the last 10 years, almost 40 GWAS-identified novel psoriasis-susceptibility loci have been identified and more recently, the genes within these loci and their significance to the pathophysiology of PV are becoming clearer [88]. Interestingly, several show clustering to a distinct segment of the inflammatory cascade [89]. Psoriasis susceptibility locus 1 (*PSORS1*), located on the MHC region on chromosome 6p21, has been most consistently identified in GWAS with a significant odds ratio of 3.0 [90]. Genes implicated within this 250 kb interval include *HLA-C* (human leukocyte antigen C), *CCHCR1* (coiled-coil α-helical rod protein 1), and *CDSN* (corneodesmosin). These were considered as potential disease-associated genes due to their function and the presence of disease-associated SNPs within their coding sequence [91]. Identification of the causal disease susceptibility allele was extremely challenging, ultimately Nair *et al.* (2006) sequenced the entire *PSORS1* region in individuals bearing different *HLA-C* alleles to identify SNPs unique to the *PSORS1* haplotype. They indicated that *HLA-Cw6* was the major *PSORS1* disease allele [92], reflecting the importance of antigen presentation in the pathophysiology of PV.

Identification of susceptibility loci has contributed to our understanding of PV pathogenesis, which appears to involve the innate and adaptive immune responses. Pathways that have been identified in various studies include IL12/IL17 axis activation (*IL23R*, *IL12B*, *IL23A*, and *TRAF31P2*), type 1 interferon induction (*IFIH1*, *RNF114*, and *TYK2*) and NF-κB signaling (*CARD14*, *REL*, *NFKBIA*, *TNFAIP3*, and *TNF1P*) [89,90,92,93,94,95,96,97]. Of particular interest is the Th1-Th17 axis, involving the recently described subset of IL17 expressing T cells (Th17) [98] which is thought to play a major role in the development and maintenance of psoriatic plaques [97].

IL12 and IL23 are cytokines that induce naïve CD4^+^ lymphocytes to differentiate into type 1 helper cells and type 17 helper cells, both of which are key mediators of PV [97]. IL12 and IL23 share a common p40 subunit encoded by the *IL12B* gene. In mice, injection of IL23 results in epidermal hyperplasia, which is mediated by IL22 produced by Th17 cells. This shows similarities to phenomena observed in humans [99]. GWAS have identified three SNPs with strong evidence of association with PV mapping near *IL12B*, *IL23A* (encoding the p19 subunit of IL23) and *IL23R* (encoding a subunit of the IL23 receptor) [94] raising the possibility that dysregulated IL23 signaling could lead to chronic immune responses within epithelial cells. Ustekinumab (Stelara^®^) is a human IgG1κ monoclonal antibody against the p40 subunit of the IL12 and IL23 cytokines that has demonstrated significant improvement in outcome measures for the treatment of PV in Phase III clinical trials [100]. A significant proportion of patients had at least 90% improvement in their psoriasis area-and-severity index (PASI) score, with a proportion experiencing complete clearance by 12 weeks [100]. These findings also establish a central role for the IL12/IL23 p40 cytokines in the pathophysiology of PV.

Another approach to utilise the discoveries gained from GWAS studies is personalised medicine. For example, patients with PV who carry risk variants in *IL12B* may benefit preferably from a monoclonal antibody targeting its p40 subunit, e.g., Ustekinumab. Studies using molecular profiling of PV and clinical phenotyping to predict treatment response have shown promise [101] and larger studies are underway. This is one example of how PV has been used as a paradigm for autoimmune disease and for proof-of-principle studies of targeted biologic therapies, because of the ease of accessing the skin and objectively measuring disease severity and responses to treatment.

Rare variants with large effect have been observed in families where PV segregates as an apparent Mendelian trait. The psoriasis susceptibility locus 2 (*PSORS2*) was first mapped in 1994 to human chromosomal region 17q25-qter in a large family of European ancestry [102]. More recently, it has been shown that *PSORS2* is due to gain of function mutations in the caspase recruitment domain family member 14 (*CARD14*) [96] using linkage analysis, targeted and exome capture in combination with NGS. On the basis of these findings, further work has uncovered rare missense variations in *CARD14* linked to PV using a large case-control study [95]. *CARD14* encodes a NF-κB activator within the skin epidermis. The mutations identified lie within the coiled-coiled domain of *CARD14* and result in enhanced NF-κB activity compared with wild-type CARD14 [95].

Generalised pustular psoriasis (GPP) can present with an acute, widespread and life-threatening eruption associated with fever and leukocytosis. It has long been considered a variant of PV. Mutations in *IL36RN*, which encodes the IL36 receptor antagonist and abrogates downstream activation of NF-κB signaling, have been shown to underlie GPP in consanguineous pedigrees of North African origin [103]. This mutation results in enhanced production of IL1, IL6, and IL8 inflammatory cytokines, which may contribute to the profound systemic inflammatory response seen clinically in these patients [103]. Similar recessive mutations in *IL36RN* have not been observed in patients with PV alone [104]. Genetic studies suggest that in fact, PV and GPP are etiologically distinct clinical entities, which consequently have important therapeutic implications [105].

## 7. Atopic Dermatitis (Eczema)

AD is a chronic inflammatory skin disease characterised by disturbed skin barrier function and dry, itchy skin. Its prevalence worldwide is increasing and in some countries affects almost 20% of children [106]. Like PV, concordance is observed in twin studies with rates of 0.72–0.86 in monozygotic and 0.21–0.23 in dizygotic twin pairs [107]. A complex interplay between environmental, genetic and immunological factors, as for many common disorders, all contribute to susceptibility and severity.

The filaggrin story is central to our understanding of AD and ichthyosis vulgaris (IV). It exemplifies how the study of a monogenic disorder can translate to a complex trait disease. In 2006, null mutations in the filaggrin gene *FLG* were first identified in Irish families with IV, which often causes dry, scaly skin and is also a strong genetic risk factor for AD [108]. Histological evidence for the possible lack of filaggrin in IV dates back to 1985 [109] however these preliminary studies were hindered by the daunting size and repetitive nature of *FLG*, particularly exon 3. The McLean group developed a successful strategy to analyse this locus with the use of long range PCR to amplify exon 3 in combination with short specific PCRs to amplify remaining overlapping fragments that were then used to reconstruct the repetitive sequence [108]. Further research has identified significant associations of *FLG* mutations with atopic asthma, allergic rhinitis and peanut allergy [110], as well as early onset and increased severity of AD [111]. These studies have been reproduced in a variety of geographical populations, including European, Japanese, Taiwanese, Chinese, and Korean [112,113,114]. Indeed, the correlation between *FLG* mutations and AD is considered one of the most robust examples of genotype-phenotype relationship in complex trait disease with an odds ratio of up to 13.4 [115].

Filaggrin plays a key role in epidermal barrier function. Briefly, its degradation products act as “natural moisturising factors” in the skin and assist the formation of a flattened granular cell layer upon keratinocyte terminal differentiation [116]. Studies describing murine models of filaggrin haploinsufficiency have shown skin barrier impairment and enhanced sensitisation to percutaneous allergens [117,118]. The significant effect of *FLG* mutations on AD risk highlights the role of impaired skin barrier function in the pathogenesis of atopic diseases. Filaggrin replacement therapies could prove significant in the management of AD. Recently, Otsuka *et al*. (2014) [119] identified a novel compound JTC801, with potential therapeutic applicability. This has been shown to increase expression of filaggrin in both human and murine keratinocytes and, when administered orally, it can hinder the development of AD-like inflammation in the NC/nga AD mouse model [119].

Although the AD spotlight has focused largely on filaggrin, several other genes have been implicated in the pathogenesis of this disorder. To date, a total of 19 genome-wide significant (*p* < 5 × 10^−8^) susceptibility loci have been identified through GWAS [120]. The first GWAS data was published in 2009 and included 939 cases and 975 controls in addition to 270 complete nuclear families with two affected siblings [121]. It identified a novel susceptibility locus in 11q13.5, located 38 kb downstream of *C11orf30*. The peak association was observed 68 kb upstream of the leucine rich repeat containing 32 gene (*LRRC32*) which has been shown to be expressed in activated human regulatory T cells [122]. Carriers have a risk of developing AD that is 1.47 times that of controls [121]. A 2011 Meta analysis of GWAS for AD included 5606 cases and 20565 controls and an additional 5419 cases and 19833 controls in a validation study [114]. Three novel risk loci reached genome-wide significance: rs479844 upstream of ovo-like zinc finger 1 (*OVOL1*), rs2164983 near actin-like 9 (*ACTL9*) and rs2897442 in kinesin family member 3A (*KIF3A*). They also confirmed association with the *FLG* locus. *OVOL1* disruption in mice leads to keratinocyte hyperproliferation and hair shaft abnormalities [93]. It is thought to play a role in regulating epidermal proliferation and loricrin expression, impairing premature terminal differentiation [123]. *KIF3A* associated SNPs map within a cluster of cytokine and immune mediated genes including Th2 cytokine genes: *IL13* and *IL4*. These cytokines have been implicated in other autoimmune and inflammatory diseases including PV [124], Crohn’s Disease [125] and asthma [125]. Increased levels of Th2 cytokines such as these have been reported in AD as well as greater levels of mRNA expression in acute skin lesions compared with unaffected skin in patients [126,127,128]. These GWAS findings highlight the role of skin barrier function (*FLG*), epidermal proliferation and differentiation (*OVOL1*) and the adaptive immune system response (*IL13-RAD50*, *LRRC32*) in the pathophysiology of AD.

Despite these promising discoveries, less than 20% of disease variance has been explained [129]. The phenomenon of “missing heritability” has been observed across other complex diseases and suggests that unmapped common and rare variants with small effect size in GWAS as well as genetic interactions may contribute to the remaining heritability [129]. Epigenetic studies focusing on the contribution of DNA and chromatin methylation may also explain the role that they play in the formation and progression of complex diseases by regulating gene expression [130]. Future work integrating GWAS and epigenetic data may provide insights into our understanding of complex trait disease. In summary, GWAS data reinforces the concept that multiple low risk variants are most likely to contribute to AD and PV, but that larger sample sizes may be necessary to identify them.

## 8. Conclusions

The post-Human Genome Project era has seen remarkable advances in our understanding of genes underlying both rare and common skin disease. Such insights have proved significant beyond the field of dermatology because of shared mechanisms of disease for example, PV and inflammatory bowel disease. The wider relevance of skin disease is highlighted by the fact that skin is frequently a marker of internal disease. For example, mutations in *ADAM17* not only cause inflammatory skin and bowel disease but increased susceptibility to infection and cardiomyopathy [131]. Similarly, the study of tylosis with oesophageal cancer, an autosomal dominant cancer syndrome that presents with skin thickening of the palms and soles, has brought to light the role of the inactive rhomboid family member iRHOM2 in cancer pathophysiology [132] and wound healing [133]. This also highlights that mechanistic studies are facilitated by the relative ease with which patient material can be obtained by skin biopsy to derive cell lines for functional studies.

Skin disease is particularly remarkable for its intragenic heterogeneity, for example distinct dominant and recessive mutations in the desmosomal Desmoplakin gene *DSP* can result in a spectrum of disease phenotypes ranging from arrhythmogenic right ventricular cardiomyopathy (ARVC) and striate palmoplantar keratoderma to palmoplantar keratoderma with woolly hair and ARVC (reviewed in [134]).

GWAS, WES and whole genome sequencing (WGS) involving increasingly larger cohorts of ethnically diverse populations may also identify additional low and high penetrance variants that contribute to phenotypic variability. WGS is becoming increasingly affordable and offers scope to become the most cost-effective method for genetic diagnostics. In parallel, advances in bioinformatics and statistics are necessary to analyse the vast quantity of data generated by these studies, and distinguish significant findings. We may also see a move towards re-classification of skin diseases and malignancies based on genome sequence and subsequently, a targeted therapeutic approach to optimise treatment outcome.
